# Paraganglioma of the Right Recurrent Laryngeal Nerve Presenting as a Thyroid Nodule

**DOI:** 10.7759/cureus.57609

**Published:** 2024-04-04

**Authors:** Amanda Raymond, Matthew N Peebles, Samantha Diaz, Wendy Rubinstein

**Affiliations:** 1 College of Medicine, Florida State University College of Medicine, Tallahassee, USA; 2 Surgery, Cleveland Clinic Florida, Port St. Lucie, USA; 3 Cancer Prevention, U.S. National Cancer Institute, Bethesda, USA

**Keywords:** neuroendocrine tumor, sdhd gene, recurrent laryngeal nerve, thyroid nodule, paraganglioma

## Abstract

Paragangliomas are abnormal growth cells of neuroectodermal origin that arise from the autonomic nervous system. Head and neck paragangliomas are rare, commonly benign and often have a hereditary origin. Head and neck paragangliomas most commonly arise in the carotid bodies, vagus and glossopharyngeal nerves, and the sympathetic chain. However, we present a case of a paraganglioma arising from the recurrent laryngeal nerve, a phenomenon that has been reported only three times before in the literature. The patient is a 49-year-old female with a past medical history of bilateral carotid body paragangliomas and Hashimoto’s disease. She has a family history of paragangliomas in her father and distant relatives and carries a pathogenic variation in the succinate-dehydrogenate subunit D (*SDHD*) gene, which was first identified through the original linkage studies involving her family. She presented with a mild swelling sensation in her neck. A thyroid ultrasound revealed a right lobe nodule measuring 3.3 x 2.2 x 2.1 cm. Fine needle aspiration of the nodule revealed an atypia of undetermined significance with a risk of malignancy judged as 50%. A total thyroidectomy was performed due to concern for malignancy. During the operation, the thyroid was nodular and hypervascular. At the right thyroid lobe, there was a pearlescent tubular structure approximately 4-5 mm in size. This was stimulated via intraoperative nerve monitoring and was consistent with being a part of the right recurrent laryngeal nerve. Pathology of the tubular structure revealed a 2.8 cm paraganglioma of the right recurrent laryngeal nerve. An incidental 0.1 cm papillary thyroid microcarcinoma within the left thyroid lobe was also noted. Our patient presented with a history of paragangliomas at a very young age and bilaterality, features that are highly characteristic of hereditary disease. Through the original linkage studies involving her family, her father was recognized as being an obligate carrier at risk of bearing occult paragangliomas. Imaging showed that he carried three paragangliomas. Identification of the familial *SDHD* syndrome as well as *SDHD* testing has now become widely available. Recognizing hereditary paraganglioma and other cancer susceptibility syndromes can help foster more knowledge on the subject and improve clinical outcomes. More attention should be put on the presentation of paragangliomas in an atypical location, such as in our case.

## Introduction

Paragangliomas are a group of neuroendocrine tumors that can arise from the parasympathetic or sympathetic ganglia. Most head and neck paragangliomas are benign, although metastasis outside the primary site varies with the underlying cause. Paragangliomas are components of known hereditary syndromes, with about 30% of them being a part of hereditary syndromes [1]. Those carrying a pathogenic variation in the succinate-dehydrogenate subunit D (*SDHD*) gene have about a 15%-29% metastatic risk [2]. The predominant anatomic sites of paragangliomas are in the head and neck, compromising about 65%-70% of all paragangliomas [3]. The most common locations for head and neck paragangliomas are the carotid body, glossopharyngeal or vagus nerves, and the jugular bulb [3,4]. However, there have only been three reports in the literature of a paraganglioma arising from the recurrent laryngeal nerve [4-6]. Thus, we present the fourth known case.

## Case presentation

**Figure 1:**
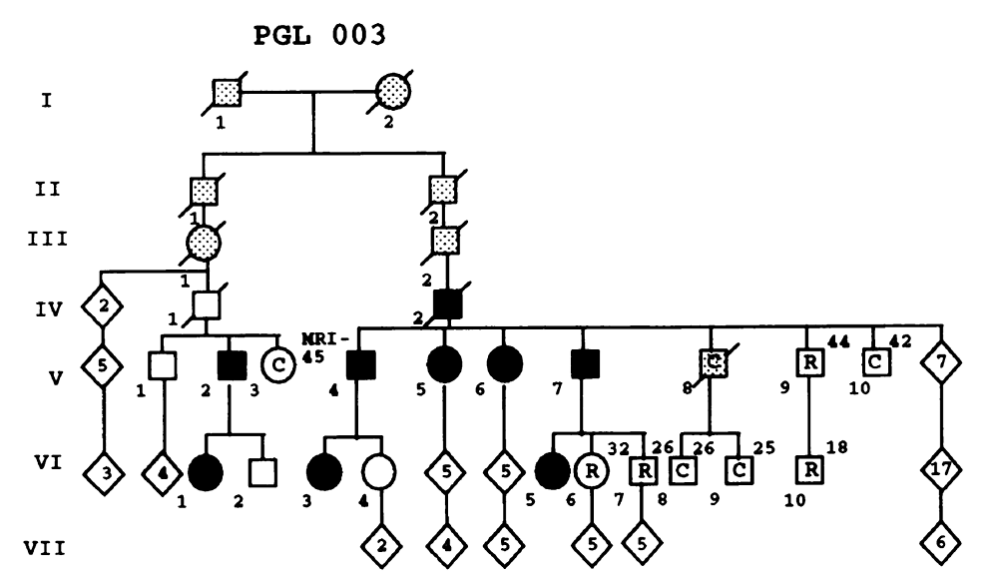
This family was one of the original pedigrees used to perform linkage analysis to identify the SDHD gene. VI-1 represents our patient. *SDHD*: succinate-dehydrogenate subunit D

**Figure 2:**
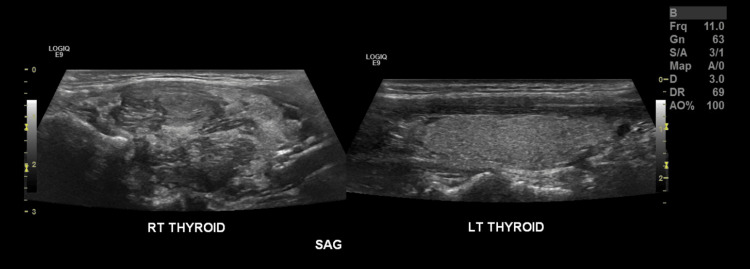
Sagittal ultrasound of the right vs left thyroid. RT THYROID: right thyroid; LT THYROID: left thyroid; SAG: sagittal

**Figure 3:**
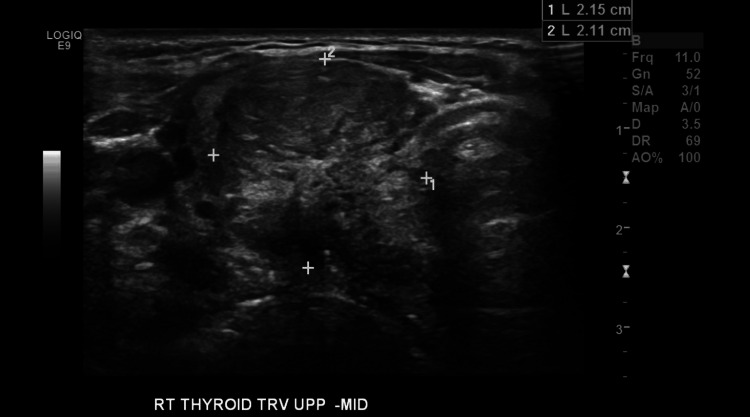
Transverse ultrasound of the right thyroid, upper-middle lobe. The ultrasound shows a 2.15 x 2.11 cm nodule. RT THYROID: right thyroid; TRV: transverse; UPP-MID: upper-middle

A 49-year-old female has a past medical history of benign paragangliomas in her neck (bilateral carotid body tumors) at age 19, along with Hashimoto's disease. She has a familial history of paragangliomas in her father and a known pathogenic variation in the *SDHD* gene (missense mutation, His^102^ → Leu^102^) (Figure 1) [7]. She is currently experiencing occasional mild pressure sensations in her neck. She reports no difficulties with swallowing or sensations of choking. She became concerned about the appearance of a mass considered to be a thyroid nodule. A thyroid ultrasound is done and reveals a very large, heterogeneous hypoechoic right lobe nodule measuring 3.3 x 2.15 x 2.11 cm (Figures 2, 3). The thyroid nodule is classified as TR4 and moderately suspicious. In the right lobe laterally, there is a 1.0 x 0.7 x 0.6 cm solid, that appears very hypoechoic, smoothly marginated nodule (Figures 2, 3). This is classified as TR4 and moderately suspicious as well. She has been initiated on a daily dosage of 25 mcg of levothyroxine (Synthroid) due to mild complaints of fatigue. The patient is referred to surgery for the abnormal thyroid ultrasound and a subsequent fine needle aspiration of the right thyroid lobe is completed. The right thyroid biopsy revealed an atypia of undetermined significance with a risk of malignancy of 50%. Since the patient was taking Synthroid at the time, a total thyroidectomy was advised as the endocrinologic effects would likely be more easily managed at a later stage.

Hospital course

A total thyroidectomy with intraoperative nerve monitoring was performed under general anesthesia. The thyroid was very firm, nodular, and hypervascular. By the right thyroid lobe, there was a pearlescent tubular structure coming up from the posterior lateral neck that extended superiorly and then dilated to approximately 4-5 mm in size while retaining its pearlescent color. This was stimulated and showed activity consistent with the right recurrent laryngeal nerve. Given the patient's history of paraganglioma within the neck, there was a concern about the potential recurrent laryngeal nerve paraganglioma, an atypical location. Both thyroid lobes were removed, and the questionable dilated portion of the right recurrent laryngeal nerve was carefully dissected free as best could circumferentially. Upon post-operation, the patient was tolerating oral intake, had no issues with swallowing, and had mild hoarseness and mild difficulty raising her voice. Pathology later revealed a 2.8 cm paraganglioma on the right recurrent laryngeal nerve as well as an incidental 0.1 cm papillary thyroid microcarcinoma confined on the left thyroid lobe.

## Discussion

Although our patient was the first person in her family to present with a paraganglioma, her extremely young age at initial presentation, coupled with bilaterality, was a feature of a hereditary disease. Her father was asymptomatic throughout his lifetime, but through pedigree analysis in the context of the original genetic linkage research study, he was recognized to likely be an obligate carrier at high risk of harboring occult paragangliomas. Upon imaging, he was demonstrated to carry three occult tumors (paragangliomas). Identification of the familial *SDHD* syndrome through the linkage study was accomplished before the completion of the Human Genome Project in 2003. Since then, *SDHD* testing has become widely available with 290 tests offered by 69 laboratories in 18 countries [8] and 690 unique variations described [9]. Nonetheless, recognition and clinical management of hereditary paraganglioma and other cancer predisposition syndromes can be further improved through awareness and education. A high level of clinical suspicion should be maintained for the presentation of paragangliomas at atypical anatomic locations, as shown in this case.

## Conclusions

This case report highlights the rare occurrence of a paraganglioma arising from the right recurrent laryngeal nerve, a phenomenon documented only three times in the literature before. This demonstrates the importance of considering atypical locations for paragangliomas, especially in patients who may have a familial genetic predisposition to them. In our case, our patient had a pathogenic variation in the *SDHD* gene, thus increasing her risk of harboring additional paragangliomas. Further research is needed to understand the underlying mechanisms and genetic factors that contribute to the development of paragangliomas in anomalous locations within the body.
